# A polymer scaffold for self-healing perovskite solar cells

**DOI:** 10.1038/ncomms10228

**Published:** 2016-01-06

**Authors:** Yicheng Zhao, Jing Wei, Heng Li, Yin Yan, Wenke Zhou, Dapeng Yu, Qing Zhao

**Affiliations:** 1 State Key Laboratory for Mesoscopic Physics, School of Physics, Peking University, Beijing, 100871, China; 2 Collaborative Innovation Center of Quantum Matter, Beijing, 100084, China

## Abstract

Advancing of the lead halide perovskite solar cells towards photovoltaic market demands large-scale devices of high-power conversion efficiency, high reproducibility and stability via low-cost fabrication technology, and in particular resistance to humid environment for long-time operation. Here we achieve uniform perovskite film based on a novel polymer-scaffold architecture via a mild-temperature process. These solar cells exhibit efficiency of up to ∼16% with small variation. The unencapsulated devices retain high output for up to 300 h in highly humid environment (70% relative humidity). Moreover, they show strong humidity resistant and self-healing behaviour, recovering rapidly after removing from water vapour. Not only the film can self-heal in this case, but the corresponding devices can present power conversion efficiency recovery after the water vapour is removed. Our work demonstrates the value of cheap, long chain and hygroscopic polymer scaffold in perovskite solar cells towards commercialization.

## Abstract (web summary)

Perovskite solar cells exhibit large conversion efficiencies, but their stability still represents a bottleneck. Here, the authors integrate a hygroscopic polymer scaffold to the perovskite active layer and fabricate efficient and stable devices that recover after being exposed to a humid environment.

## Graphical abstract

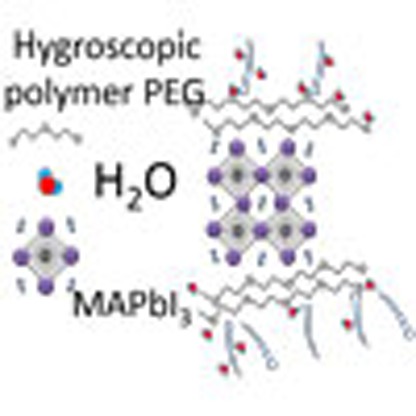

The rapid rising of the lead halide perovskite solar cells (PSCs), typically based on CH_3_NH_3_PbI_3_ (MAPbI_3_) or its analogues, demonstrate great potential for market applications^1^,^2^,^3^,^4^,^5^,^6^,^7^,^8^,^9^,^10^,^11^,^12^,^13^. The worldwide power conversion efficiency (PCE) race for the perovskite solar cells has stimulated the highest record of >20% update via solvent engineering^8^, interface engineering^5^ and composition engineering^10^, which approaches the best of polycrystalline silicon modules. The excellent performance of PSCs is attributed to the high absorption coefficient, weak exciton binding energy, and long diffusion length of the perovskite phase^12^,^14^,^15^,^16^,^17^. Two dominant device architectures, namely mesoporous scaffold^1^,^2^,^3^ and planar heterojunction structures^4^,^5^ have been developed. The PCE in mesoporous scaffold architecture depends on the pore size, porosity and morphology of the metal oxide nanoparticles, which dominantly determine the coverage, morphology variation of the perovskite layer and the carrier lifetime. Moreover, the fabrication of inorganic metal oxide mesoporous scaffold is complicated and needs sintering at high temperature of >450 °C (refs 2, 3), which makes the fabrication more expensive. The planar heterojunction PSCs fabricated layer by layer is either fabricated via high vacuum deposition^4^ or solution-based method^5^,^18^. However, the low-cost solution process is rather challenging to form homogeneous film due to the dewetting process and sensitivity to the atmosphere^18^,^19^,^20^. Recently, progress has been reported to control the nucleation rate by changing dissolvent or temperature of the substrate^21^,^22^. Besides high efficiency and high reproducibility, long-term stability is also a crucial barrier to commercialize the PSCs, because the lead halide perovskite materials are very sensitive to ambient humidity and easy to dissolve and degrade in humid environment^5^,^23^,^24^. Therefore, it is very challenging and pressing to develop ultra stable and high PCE devices resistant to hostile operation environment.

Here we report a novel perovskite solar cell architecture based on an insulating polymer scaffold structure. The polymer-scaffold perovskite layer is simply fabricated via a single one-step process under mild temperature of 105 °C. The as-made polymer-scaffold perovskite solar cell (PPSC) devices demonstrate high PCE up to ∼16% under standard AM1.5 illumination and very small efficiency variation compared with that without the insulating polymer. Remarkably, unsealed PPSCs exhibit excellent performance in stability test in highly humid environment (70% relative humidity), which can endure the moisture for over 300 h. Most strikingly, the PPSCs show strong self-healing or water resistant effect under vapour spray. The perovskite film, as well as the corresponding devices can recover rapidly to its original phase and performance after being vapour sprayed and dried in ambient air, respectively.

## Results

### Design, fabrication and characterization of PPSCs

**Figure 1:**
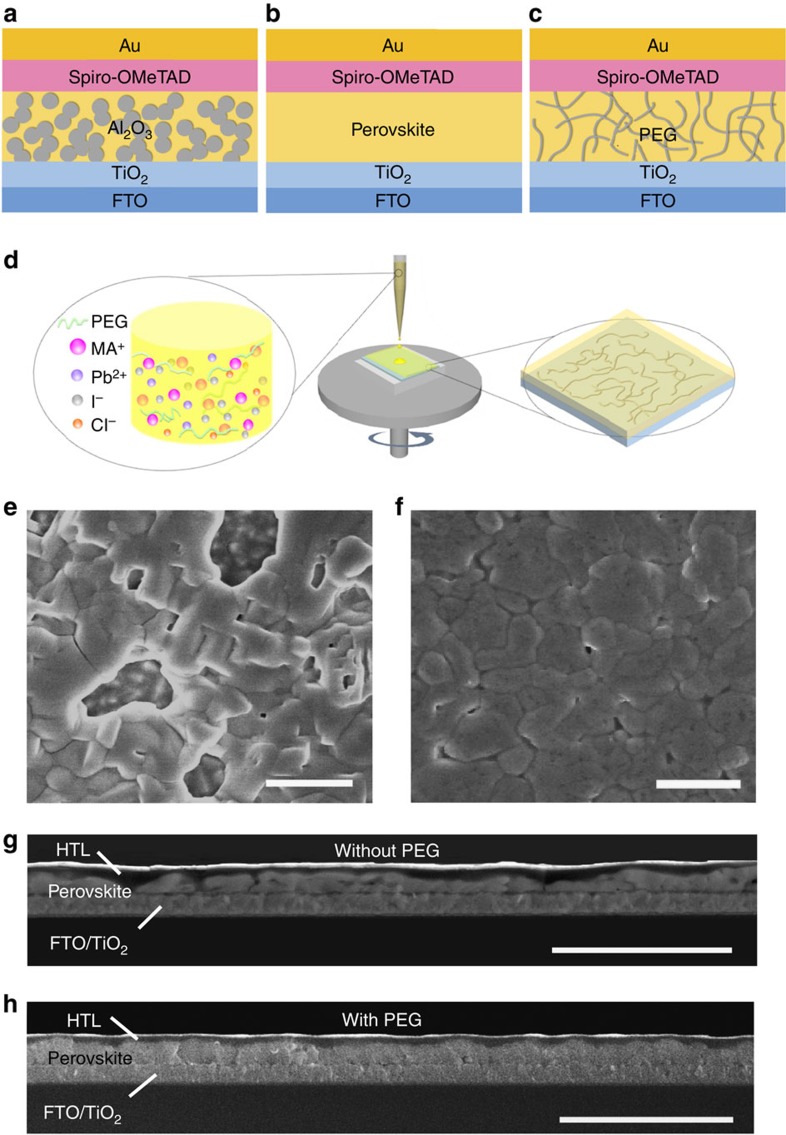
Scheme and morphology of polymer-scaffold perovskite solar cells. Schematic diagram of mesoporous-scaffold-structured PSC (**a**), planar-heterojunction-structured PSC (**b**) and polymer-scaffold structured PSC (**c**). (**d**) Schematic diagram showing the fabrication process of perovskite film with polymer scaffold using one-step spin coating method. (**e**,**f**) Top-view SEM images of the perovskite films (**e**) without PEG and (**f**) with PEG. Scale bar, 1 μm. (**g**,**h**) Cross-sectional SEM images of the perovskite solar cells (**g**) without PEG and (**h**) with PEG scaffold. Scale bar, 3 μm.

The PPSC architecture inherits the basic structure of PSCs with inorganic mesoporous scaffold^1^,^2^,^3^, instead, the inorganic nanocrystal scaffolds are replaced with long- and flexible-polymer chain networks, which can be processed under mild temperature (Fig. 1a–c). The fabrication of PPSC is similar to that of planar heterojunction PSCs, except for the precursor solution preparation (Fig. 1d). Long-chain insulating polymers, dissolved in precursor solution, assemble the polymer scaffold simultaneously as the precursor solution is spin coated on the substrate. The selected polymer should be chemically inert to ingredients for perovskite synthesis; it should also act as an electrical insulator to guarantee the effective transport of photo-generated carriers. Here polyethylene glycol (PEG), which meets the requirements, is chosen to be added in the precursor solution of PbCl_2_ and CH_3_NH_3_I (MAI; 1:3 molar ratio) in dimethylformamide. The molar ratio of PEG monomers (C_2_H_4_O) to ultimate product MAPbI_3_ is close to 1:1 (PEG in 40 mg ml^−1^). The PEG scaffold perovskite absorber layer was prepared on the TiO_2_ (∼40 nm thickness)-coated fluorine doped tin oxide (FTO) glass by spin coating followed by annealing at 105 °C for 70 min. 2,29,7,79-tetrakis-(N,N-di-p-methoxyphenylamine)9,99-spirobifluorene (Spiro-OMeTAD) and gold were used as the hole transport layer and electrode, respectively.

Figure 1e,f present top-view scanning electron microscope (SEM) images of the perovskite films without and with polymer scaffold. Those without scaffold exhibited large pinholes and bare conducting substrates, increasing the risk of short circuits. Those with scaffold demonstrated continuous and complete coverage. The cross-section SEM image showed the perovskite layer without polymer to have considerable variation in thickness and many voids (Fig. 1g). The film with polymer was uniform in thickness over large area (Fig. 1h). The PEG scaffold can also work in another precursor solution PbI_2_:MAI (1:1) (Supplementary Fig. 1). PEG plays an important role in improvement of the film quality for the following reasons. First, the simultaneously formed PEG scaffold, which is formed by entangled long-chain molecules, acts as a three-dimensional skeleton to support perovskite crystals, causing it to cover the substrate more uniformly (Fig. 1c), demonstrating an advantage over planar heterojunction PSCs. Uniformly distributed PEG scaffolds function as Al_2_O_3_ scaffolds^3^, and they can be produced in a much simpler and less costly manner. Second, the wetting properties of the precursor solution have been improved after introducing PEG (Supplementary Fig. 2), which can improve film morphology^19^. Third, PEG molecules in precursor solution can slow down the crystallization of perovskite from the X-ray diffraction characterization by tracking the intermediate phase in the crystallization process, which is shown below.

**Figure 2:**
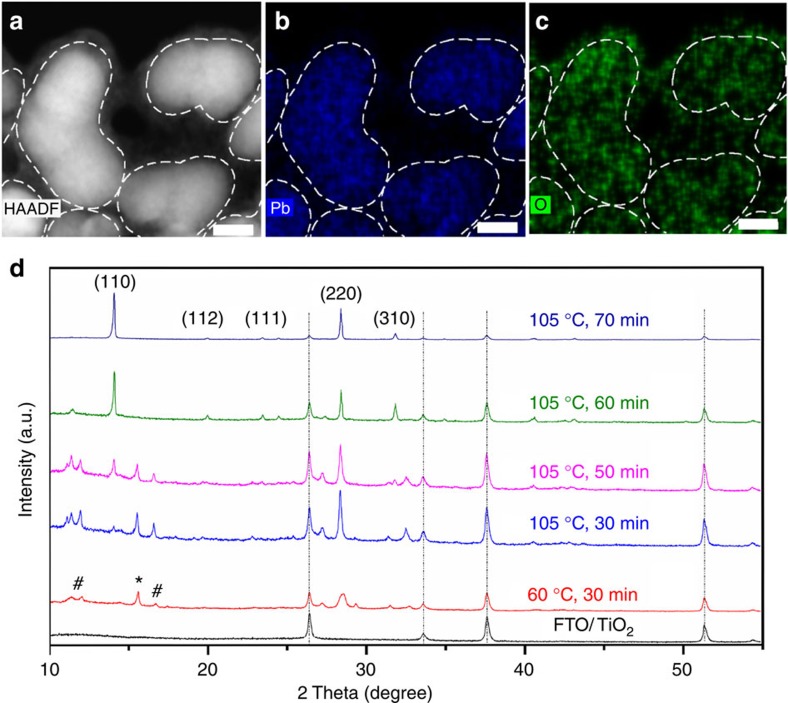
Characterization and structure evolution of polymer-scaffold solar cells. (**a**) HAADF image of the perovskite film with PEG scaffold in STEM mode. Scale bar, 10 nm. (**b**) EDS mapping of Pb element of the peroskite film. Scale bar, 10 nm. (**c**) EDS mapping of O element of the peroskite film. O signal corresponds to PEG distribution because only the PEG contains O element among the materials used in perovskite thin film synthesis. Scale bar, 10 nm. (**d**) X-ray diffraction patterns of the perovskite film (PEG molecule weight: 20,000 Da; PEG concentration: 40 mg ml^−1^) evolution with annealing time. ‘#' symbols and ‘*' symbols denote the intermediate phase and MAPbCl_3_ phase, respectively.

To confirm that PEG acts as scaffold in the architecture, detailed energy dispersive spectroscopy (EDS) analysis in a Cs-corrected scanning transmission electron microscope (STEM) was conducted for element mapping investigation (Fig. 2a–c). The high-angle annular dark field (HAADF) STEM image (Fig. 2a) reveals that the perovskite crystalline grains are surrounded by polymer material. The profiles of perovskite grains are highlighted with dashed lines according to the EDS mapping of Pb component (Fig. 2b). The amorphous material surrounding the perovskite grains is PEG in accordance with the EDS mapping of O element because only PEG contains O. The O mapping demonstrates that PEG not only covers the perovskite grains, but also cross-links the neighbouring perovskite grains via self-assembly forming a polymer-scaffold network to support the perovskite grains (Fig. 2c). Above conclusion is also evidenced by the fact that O mapping covers larger area than Pb and extends from crystal boundaries, indicating that the perovskite grains are imbedded in the PEG networks, corresponding well to the HAADF–STEM image. In addition, the O element mapping on the cross-sectional area of the PPSC film confirms a homogeneous distribution of PEG molecules in the perovskite film (Supplementary Fig. 3). High-resolution transmission electron microscope image of perovskite grain demonstrates good crystal lattice (Supplementary Fig. 4), suggesting that PEG molecules do not damage the perovskite crystal structure.

The phase evolution of PEG scaffold perovskite film during annealing was characterized by X-ray diffraction analysis (Fig. 2d). Over the course of 30–50 min, the film turned from orange to yellow, the (110) and (220) peaks of perovskite phase^25^,^26^ became more pronounced and some new peaks appeared between 10 and 20°. The intermediate phase disappeared after annealing for 60 min, and the sample became deep black at 105 °C after 70 min of annealing, with stronger (110) and (220) peaks. X-ray diffraction patterns of perovskite film without PEG and with PEG under the same 100 °C sintering temperature are also shown in Supplementary Fig. 5. Note that intermediate phase disappears after 70 min in film without PEG. While in film with PEG, intermediate phase disappears after 90 min (Supplementary Note 1), which strongly suggests a slower crystallization process in PEG scaffold perovskite film.

**Figure 3:**
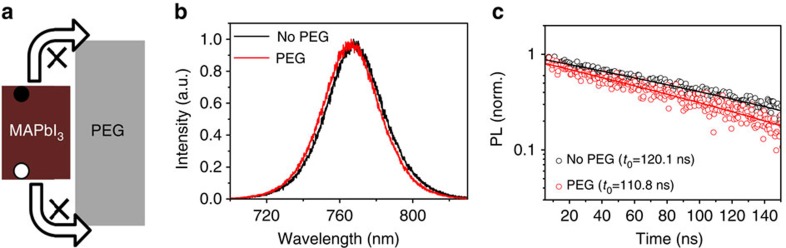
Photoluminescence and energy-level alignment of polymer-scaffold perovskite films. (**a**) Schematic diagram of the energy offsets between CH_3_NH_3_PbI_3_ and PEG. (**b**) PL spectra of pristine perovskite film (control) and the corresponding perovskite film after being coated with PEG. (**c**) Characteristics of PL transient spectra of PEG scaffold perovskite films and perovskite film without PEG, indicating the similar lifetime of carriers in perovskite with and without PEG scaffold.

Photoluminescence (PL) spectra were measured to verify whether excess recombination sites and defect states are induced in the PEG scaffold perovskite film^27^,^28^. Compared with the PL spectrum of pristine perovskite film, the intensity is not influenced after being coated with PEG (Fig. 3b), indicating the insulating nature of PEG, as shown in Fig. 3a. Figure 3c shows the time-resolved PL spectra with and without PEG polymer scaffold, with the similar carrier lifetime by using single exponential fitting (110.8 ns with PEG, 120.1 ns without PEG). This result indicates ∼1 μm diffusion length if diffusion coefficient is supposed to be 0.1 cm^2^ s^−1^, which is sufficient to guarantee effective collection efficiency. On the other hand, no visible shift (peak position of 760 nm) and broadening (half width of 40 nm) of peaks associated with perovskite layers with and without PEG scaffold is observed, indicating that PEG does not induce any excessive recombination (Supplementary Fig. 6).

### Photovoltaic performance of PPSCs

**Figure 4:**
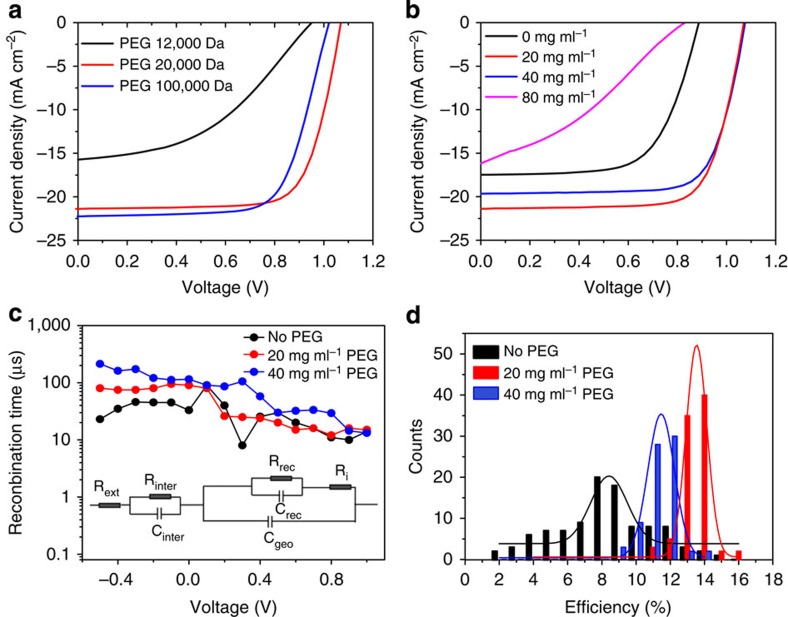
Photovoltaic characterization of polymer-scaffold perovskite solar cells. (**a**) *J*–*V* curves of the reverse scan based on precursor solution with different PEG molecular weight (concentration: 20 mg ml^−1^) measured from 1.5 to −0.2 V under 500 mV s^−1^. (**b**) *J*–*V* curves of the reverse scan based on precursor solution with different PEG concentration (molecular weight: 20,000 Da) measured from 1.5 to −0.2 V under 500 mV s^−1^. (**c**) Recombination time constant *R*_rec_.*C*_rec_ (*R*_rec_, recombination resistance, *C*_rec_, capacitance) under different applied voltage can be extracted by fitting the Nyquist plot using the inset circuit. (**d**) PCE distribution of perovskite solar cells with (20 or 40 mg ml^−1^) and without PEG scaffold. PCE values are extracted from reverse scan curve measured from 1.5 to −0.2 V under 500 mV s^−1^. The active area for testing is 9 mm^2^.

The photovoltaic properties of PPSCs are characterized systematically. The PEG molecular weight and its concentration in precursor solution were found to play an important role in the performance of PPSCs. Figure 4a shows the current density–voltage (*J*–*V*) curves of samples with PEG in different molecule weights (12,000/20,000/100,000 Da) measured under simulated AM1.5 illumination of 100 mW cm^−2^. The molecular weight of PEG, proportional to the length of polymer, has a significant effect on the performance of PPSCs, and the optimal molecular weight was found to be >20,000 Da because longer PEG molecules facilitate the formation of three-dimensional molecular network in the perovskite film, and guarantee uniform morphology. PEG concentration is also critical to devices because it is related to the series resistance. As demonstrated in Fig. 4b, the device shows much better performance with 20 and 40 mg ml^−1^ PEG concentration, and its PCE decreases at higher concentrations (80 mg ml^−1^) because the excess PEG in the film increases the series resistance and lowers the efficiency. Note that viscosity of the solution can be strongly influenced by PEG concentration and molecule weight, so the r.p.m. value of spin coating is optimized at different PEG concentration and molecule weight (see Methods for details) to guarantee ∼400 nm film thickness. The improved efficiency was also consistent with the increased recombination time constant^28^ in PPSCs, compared with that of control devices, as is shown in Fig. 4c. Owing to the improved coverage, the recombination time is almost one order of magnitude larger than that without polymer scaffold at zero bias. The highest PCE of 16% was obtained for PPSCs with optimal PEG molecule weight of 20,000 Da and concentration of 20 mg ml^−1^, and showed an open voltage (*V*_oc_) of 0.98 V, short-circuit current (*J*_sc_) of 22.5 mA cm^−2^, and fill factor of 0.72 (Supplementary Fig. 7a). Another representative device shows a steady-state PCE of 15.4% at first 10 s, and the PCE gradually drops down and eventually stabilized at 13.5% in 1,000 s (0.7 V bias, Supplementary Fig. 7b). Parallel devices present *H* value from 10 to 30% under 500 mV s^−1^ if hysteresis index is defined *H*=(PCE_reverse_−PCE_forward_)/PCE_reverse_ × 100% to characterize the hysteresis effect. PCE_reverse_ and PCE_forward_ represent PCE obtained from *J* to *V* measurement in reverse scan and forward scan, respectively. The statistical PCE distributions of >100 samples without and with (for two concentrations) polymer scaffolds are shown in Fig. 4d. The PPSCs exhibited a narrow PCE distribution for 20 and 40 mg ml^−1^, with an average efficiency of 14 and 12.5%, respectively, but the PCE distribution of the PSCs without polymer scaffold was much broader, varying from 2 to 14%, averaging at ∼8%. The significant increase in PCE and high reproducibility of the PPSC devices are direct reflections of the uniform thickness and homogeneous morphology of the polymer-scaffold perovskite layer.

### Improved stability in highly humid environment

**Figure 5:**
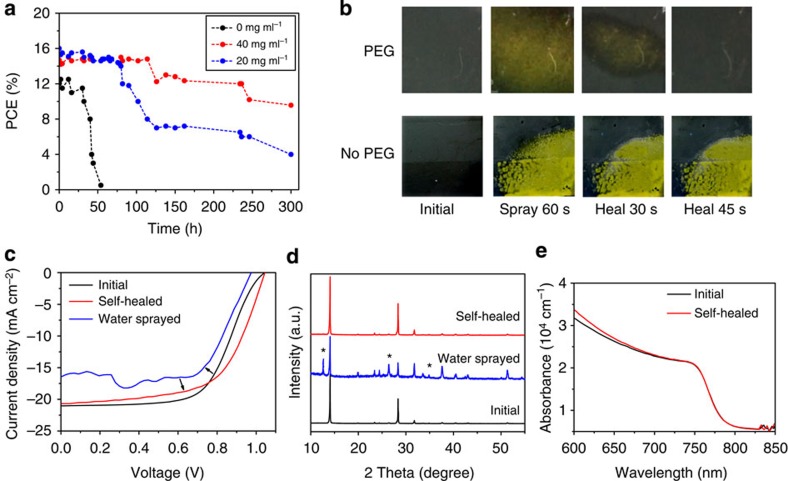
Device stability and self-healing demonstration. (**a**) PCE evolution as a function of time of perovskite solar cells with 20 or 40 mg ml^−1^ PEG and without PEG scaffold exposed in high humid (70% relative humidity) dark environment without any sealing. (**b**) Photographs of perovskite films with and without PEG showing colour change evolution after water-spraying for 60 s and kept in ambient air in 45 s. (**c**) *J*–*V* curves of PPSCs before and after water spray, revealing a complete recover of the cells in one minute when it puts back to ambient air. (**d**) X-ray diffraction evolution revealing the self-healing process: X-ray diffraction pattern of initial perovskite film with PEG, after vapour sprayed and after 10 min from removing from water vapour, respectively. The symbol ‘*' represents for the peaks of PbI_2_. (**e**) Absorption coefficient as function of wavelength for perovskite film before and after vapour spray and self-healing.

More importantly, the insulating PEG scaffold structure can stabilize the PSCs in humid environment due to the strong hygroscopicity property of PEG. Here three groups of unsealed devices were prepared without and with PEG (two concentrations), respectively. Figure 5a shows the PCE evolution as a function of time of the three groups of devices in the dark under highly humid environment (relative humidity 70%) without any sealing. In contrast to the fast PCE decay from 12 to 0.5% within 50 h of the pristine PSCs, the PPSCs retained 65% of its PCE after 300 h aging. Similarly, the *J*_sc_, *V*_oc_, and fill factor of the pristine sample decreased quickly during the first 50 h, while those of the PPSCs retained relatively high values for up to 300 h (Supplementary Fig. 8a,b). Note that the device with PEG concentration of 40 mg ml^−1^ showed better performance than the one with 20 mg ml^−1^ because more PEG can form a more compact layer, enhancing the moisture resistance of devices. We also studied the stability under continuous light illumination in 70% humidity for unsealed devices (Supplementary Fig. 8c,d). Photovoltaic performance degraded quickly within 10 h in the device without PEG scaffold, while PPSC retained 83% of its PCE during the same aging period. Similarly, *V*_oc_, *J*_sc_, and fill factor suffered substantial decrease in the first 10 h for pristine sample while they remained relatively high values in the presence of PEG. X-ray diffraction patterns of the perovskite film with PEG scaffold showed no structure destruction after 72 h continuous light irradiation, while the samples without PEG degraded into PbI_2_ quickly within 10 h (Supplementary Fig. 9). Evidence above strongly suggests that PEG can greatly protect perovskite film from decomposition under moisture attack and significantly improve the stability of PPSCs at very humid environment.

### Self-healing property of PPSCs after vapour spray

In addition to its improved stability, the PPSC devices can demonstrate a self-healing behaviour and humidity resistance effect on exposure to water vapour. Figure 5b shows a comparison of the changes in colour of the perovskite layer with PEG (top) and the perovskite layer without PEG (bottom) after both were sprayed with water vapour for 60 s. The perovskite film without PEG decomposed into PbI_2_ and turned yellow irreversibly. In contrast, the PEG scaffold perovskite film showed yellow in colour at first and recovered to black in ∼45 s after removing from the spray (Fig. 5b). This amazing self-healing behaviour is further vividly demonstrated in 120 s videos (Supplementary Movies 1 and 2). The self-healing capability of the PPSC device was also manifested in the *J*–*V* characterization (Fig. 5c). The PCE degrades with smaller *V*_oc_ and *J*_sc_ after exposure to water vapour for 60 s. Surprisingly, results showed that the *J*–*V* curve can almost regain its original value in 45 s, indicating that the PPSCs can heal itself when it is returned to ambient air after being sprayed with water. This unique humidity-resist characteristic is very suitable for practical applications because once the devices are exposed to very humid environment; they can self-heal to high PCE again in a short time when they return to sunlight again. Moreover, to further prove the self-healing property, perovskite film with PEG was sprayed with water vapour for 60 s, then after self-healing, it was coated by Spiro-MeOTAD and Au electrode to fabricate into corresponding device, which showed photovoltaic performance comparable to its original PPSC devices without water spray (Supplementary Fig. 10).

The self-healing process was recorded by X-ray diffraction analysis (Fig. 5d). The peak at 12.7° reveals that PbI_2_ phase formed when water vapour was sprayed on the film. After self-healing, the PbI_2_ peak disappeared and the perovskite film fully recovered to its original crystal phase. The self-healing can also be demonstrated by the absorption spectra (Fig. 5e), which presented the same absorbance and similar Urbach energy (∼40 meV; ref. 29). The decomposition of perovskite phase is caused by the chemical reaction with water molecules^30^,^31^,^32^, which is manifested by the X-ray diffraction analysis of the perovskite film without PEG after water spray (Supplementary Fig. 11).

**Figure 6:**
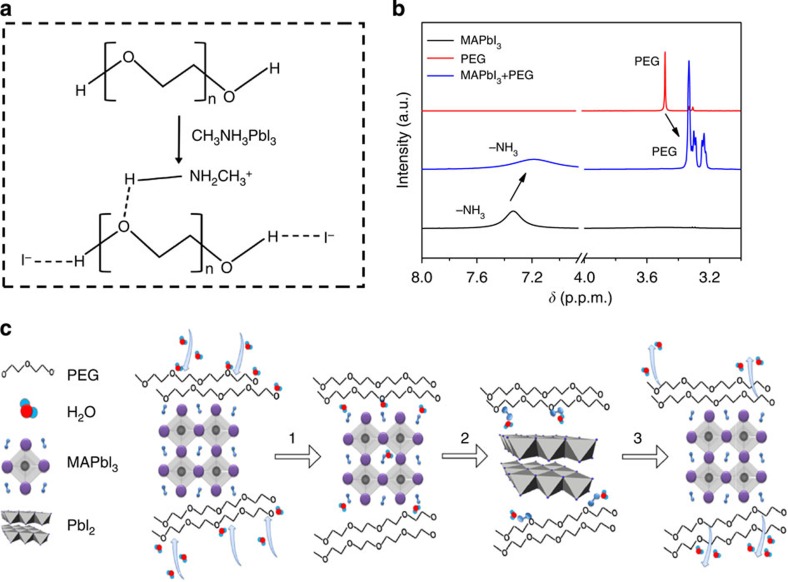
Mechanism demonstration of self-healing. (**a**) Schematic diagram of the hydrogen bonding formation between PEG molecules and MAPbI_3_. (**b**) A comparison of NMR spectra from 3.0 to 8.0 p.p.m. among three samples: deuterated DMSO solutions with MAPbI_3_, mixture of MAPbI_3_+PEG and PEG, respectively. (**c**) Schematic diagram to show mechanisms for the self-healing properties in PPSCs: (1) Water absorb on perovskite; (2) Perovskite hydrolysis into PbI_2_ and MAI·H_2_O by water; (3). Restrained MAI by PEG react with nearby PbI_2_ to form perovskite again after water evaporates. PEG has a strong interaction with MAI, preventing it from evaporating, subsequently MAI and PbI_2_ react *in situ* to form perovskite after the film was removed away from the vapour source.

According to the observations in Fig. 2a–c, we conjecture that these entangled PEG molecules are anchored on the surface of perovskite grains by hydrogen bonding, and the formation is illustrated in Fig. 6a. The –OH I–interaction had been evidenced in the work by Li *et al*^32^. We use liquid state ^1^H NMR measurements to prove such interactions. The proton NMR spectra of three samples were compared (Fig. 6b): deuterated DMSO solution with MAPbI_3_ (sample 1), with MAPbI_3_+PEG (sample 2) and with PEG (sample 3). In the signals of sample 3, double methylene group linked to oxygen on both ends (–[O–CH_2_–CH_2_]_*n*_–) is characterized by the peak at *δ*=3.48 p.p.m. (Fig. 6b). An upfield chemical shift of Δδ ∼−0.18 p.p.m. with several splitting peaks is observed in sample 2. Such chemical shift can be explained by the hydrogen bond between MA^+^ and O in PEG chain, which weakens the influence of O on protons of methylene in –[O–CH_2_–CH_2_]_*n*_–. Furthermore, the proton resonance signals of –NH_3_^+^ in sample 1 (peak at *δ*=7.33 p.p.m.) shift towards upfield with Δδ∼ −0.13 p.p.m. in sample 2 (Fig. 6b), which can also be attributed to the hydrogen bonds discussed above. Such interaction is also evidenced by a dissolution experiment devised by us (Supplementary Fig. 12).

As presented in Fig. 6c, the improved stability and self-healing effect of the PPSC devices can be ascribed to the excellent hygroscopicity of the PEG molecules and their strong interaction with the perovskite. On one hand, the omnipresent PEG molecules can absorb water efficiently to form a compact moisture barrier around perovskite crystal grains with little water penetrating into the film. On the other hand, on water spray, the black perovskite film with PEG turned light yellow (PbI_2_ forms) at first. However, due to the strong interaction between MAI and PEG, (NMR measurements and a dissolution experiment in Supplementary Fig. 12), the MAI molecules were anchored by the nearby PEG molecules rather than escape away (process 1, 2 in Fig. 6c). After being kept away from water vapour, PbI_2_ in the film reacted with nearby MAI to regenerate the perovskite MAPbI_3_ phase, very similar to the two-step synthesis^2^ (process 3 in Fig. 6c). The instant decomposition-regeneration mechanism explains the fast self-healing process in the PEG scaffold perovskite film.

## Discussion

The hygroscopic PEG scaffold can stabilize the perovskite film, rendering the devices resistant to moisture with strong self-healing property. Hygroscopic polymer-scaffold architecture paved a new effective way in perovskite solar cell, to solve the hydrolysis problem in ambient air with greatly decreased package cost. The polymer-scaffold perovskite layer reinforces the architecture and improves strain tolerance. It may also facilitate production of flexible, wearable devices in the future. Future work will be focused on looking for cheap long-chain polymers which have stronger hygroscopicity and binding effect with MAI to protect the perovskite from decomposition in ambient environment. Moreover, by comparing the stability test result under light illumination (Supplementary Fig. 8) and in dark (Fig. 5a), we found that light has a strong destructive influence on stability performance. One would expect that this influence comes from decomposition of perovskite film accelerated by light with the aid of hydrolysis by water molecules under illumination (reactive equation in Supplementary Fig. 11). However, after 72 h light soaking, the perovskite film with PEG was not destructed compared with that without PEG from X-ray diffraction spectra (Supplementary Fig. 9). This indicated another important factor influencing the efficiency in addition to material decomposition. Hence, we speculate that I^−^ ion migration^33^,^34^,^35^,^36^ may be induced or enhanced by light. Ion migration may answer for the poor stability under illumination, no matter in the traditional architecture without PEG scaffold or in our PPSCs, which needs further investigation. Further improvement needs be focused on revealing the mechanism of ion migration in the long-term stability issue, as well as how to inhibit it in PPSCs, which would lead to much better performance in stability test under illumination with high humidity.

## Methods

### Device fabrication and device characterization

All chemicals were purchased from Sigma-Aldrich or J&K Scientific Ltd. unless otherwise stated. The photovoltaic devices were fabricated on FTO-coated glass (Pilkington, Nippon Sheet Glass). First, laser-patterned, FTO-coated glass substrates were cleaned by ultrasonic cleaned in deionized water, acetone and ethanol, followed by an ultraviolet treatment for 5 min. Compact layers were deposited on the substrates by spin coating titanium diisopropoxide bis(acetylacetonate) solution (75% in 2-propanol) diluted in ethanol (1:20, volume ratio) for two times and annealed at 450 °C for 30 min. After cooling to room temperature, the substrates were transferred to a hot plate at 90 °C before spin coating. CH_3_NH_3_I was synthesized according to the reported procedure^1^. In a typical synthesis, 33.77 ml methylamine (33% in methanol) and 30 ml of hydroiodic acid (57% in water) were reacting in a 250 ml flask at 0 °C for 2 h under stirring. The precipitate was formed by evaporation at 50 °C for 1 h. The product, methylammonium iodide CH_3_NH_3_I, was washed with diethyl ether by stirring the solution for 30 min, repeated three times, and then finally dried at 60 °C in vacuum oven for 24 h. The prepared CH_3_NH_3_I, PbCl_2_ (Aldrich) (3:1) for 430 and 250 mg ml^−1^ solution was mixed with PEG (Sigma-Aldrich) and stirred in dimethylformamide at 60 °C for 12 h. The molar ratio of PEG monomers (C_2_H_4_O) to ultimate product MAPbI_3_ is ∼1:1 (PEG in 40 mg ml^−1^). The resulting solution was then coated onto the TiO_2_/FTO substrate by spin coating at 500 r.p.m. for 10 s, and then 3,500/4,500/5,500 r.p.m. for 30 s, for the solution with 20/40/80 mg ml^−1^ PEG, respectively. For different molecular weight 12,000/20,000/100,000 Da. with 20 mg ml^−1^, we use the optimized 3.500/3.500/4.500 r.p.m. to prepare the film. Then the substrate was dried on a hot plate at 60 °C for 45 min, then sintered at 105 °C for 70 min. For the preparation of peroskite film without PEG, the optimized sintering temperature is 100 °C. After cooling to room temperature, the hole transport material was spin coated onto the perovskite film at 3,000 r.p.m. for 40 s. The spin coating formulation was prepared by dissolving 72.3 mg 2,2′,7,7′-Tetrakis(N,N-p-dimethoxy-phenylamino)-9,9′-spirobifluorene(spiro-MeOTAD), purchased from Yingkou OPV Tech New Energy Co. Ltd., 30 μl 4-tert-butylpyri-dine and 20 μl of a stock solution of 520 mg ml^−1^ lithium bis (trifluoromethylsulphonyl) imide in acetonitrile in 1 ml chlorobenzene. Finally, 90-nm-thick gold electrodes were deposited on top of the devices by evaporation at ∼10^−4^ mbar. The active area of the electrode was fixed at 9 mm^2^.

The surface morphology and EDS mapping of the perovskite thin film was characterized by SEM (Nano430, FEI). The instrument uses an electron beam accelerated at 15 kV, enabling operation at a variety of currents. Considering that only PEG polymer contains oxygen element among those materials used in perovskite thin film synthesis, here oxygen stands for PEG polymer, lead for perovskite. HAADF–STEM is examined by Cs-corrected FEI (Titan G2 80–200) transmission electron microscope operating at an accelerating voltage of 300 kV, equipped with ChemiSTEM EDS detector, which enables very quick high-resolution element mapping. The sample is prepared from perovskite precursor solution spin coated on TEM grid (3,000 r.p.m.), then annealed at 105 °C for 70 min.

For NMR measurement, it is examined by Bruker 600 UltraShield using pulse signal. We use deuterated DMSO as solvent. The CH_3_NH_3_PbI_3_ solution is prepared from CH_3_NH_3_I, PbI_2_ (Aldrich; 1:1) for 53 and 153 mg ml^−1^ solution and stirred in DMSO at 60 °C for 12 h. CH_3_NH_3_PbI_3_ solution mixed with PEG (Sigma-Aldrich) is prepared by adding 40 mg PEG (molecule weight: 20,000 Da) to 1 ml CH_3_NH_3_PbI_3_ solution.

For X-ray diffraction measurement, flat PbI_2_ and TiO_2_/PbI_2_ nanocomposites were deposited on glass slides using the above-mentioned procedures. X-ray powder diagrams were recorded on an X'PertMPD PRO from PANalytical equipped with a ceramic tube (Cu anode, *λ*=1.5406 Å), a secondary graphite (002) monochromator and a RTMS X'Celerator detector, and operated in BRAGG-BRENTANO geometry. The samples were mounted without further modification, and the automatic divergence slit and beam mask were adjusted to the dimensions of the thin films. A step size of 0.008 deg was chosen and an acquisition time of up to 7.5 min per deg. A baseline correction was applied to all X-ray powder diagrams to remove the broad diffraction peak arising from the amorphous glass slide.

Steady-state PL spectra were measured using a He–Cd laser (325 nm in wavelength) and green laser (514 nm in wavelength) guided by a micro-zone confocal Raman spectroscope (Renishaw inVia microRaman system) as the laser beam with a spot size diameter of 2 μm. The collected duration is ∼5 ms. The time-resolved fluorescence spectra were recorded with a high-resolution streak camera system (Hamamatsu C10910). We used an amplified mode-lock Ti: Sapphire femtosecond laser system (Legend, Coherent) and a two-stage optical parametric amplifier (OperA Solo, Coherent) to generate the pump beam with a repetition rate of 1 KHz. All the samples were excited by 517 nm at room temperature with 110 nJ cm^−2^.

The electrochemical impedance spectrum and cyclic voltammograms were measured using a potentiostat/galvanostat (SP-150, Bio-Logic, France). The frequency can be tuned from 0.1 Hz to 1 MHz. All the samples were measured under 10 mW cm^−2^.

The *J*–*V* characteristics were obtained using an Agilent B2900 Series precision source/measure unit, and the cell was illuminated by a solar simulator (Solar IV-150A, Zolix) under AM1.5 irradiation (100 mW cm^−2^). Light intensity was calibrated with a Newport calibrated KG5-filtered Si reference cell. We use black mask to define the cells' area, and the masking effect is confirmed by testing the *J*_sc_ with and without it, which has a 5% *J*_sc_ difference. The *J*–*V* curves are tested from 1.5 to −0.2 V with a scan velocity 500 mV s^−1^. The masked active area is 9 mm^2^.

### Stability test

Unsealed PPSCs and PSCs were put near a humidifier to control its relative humidity ∼70%. For the stability test under dark, the cells were stored in a black box with 70% relative humidity, and were tested every 8 h. For the stability tests under continuous light illumination, light source ranges from 300 to 800 nm wavelength with 70 mW cm^−2^ was used. *J*–*V* curves were recorded under AM1.5 light irradiation. The temperature is controlled ∼30 °C. Three devices were measured in this way and demonstrate excellent repeatability. For the self-healing behaviour, we sprayed the water vapour for 60 s onto the resulting devices by humidifier. *J*–*V* curves were collected before and right after water vapour exposure under standard illumination AM1.5.

## Additional information

**How to cite this article:** Zhao, Y. *et al*. A polymer scaffold for self-healing perovskite solar cells. *Nat. Commun*. 7:10228 doi: 10.1038/ncomms10228 (2016).

## Supplementary Material

Supplementary Information Supplementary Figures 1-12 and Supplementary Note 1

Supplementary Movie 1 Real time videos of the perovskite film without PEG exposed to humidifier for about 60 sec and the color change after that in 1 min.

Supplementary Movie 2 Real time videos of the perovskite film with PEG exposed to humidifier for about 60 sec and the color change after that in 1 min. Strong self-healing behavior of perovskite film with polymer scaffold was demonstrated. The perovskite film with polymer scaffold can be self-healed after 1 min accompanied by the color change turning from yellow to black again.
