# Supplementary material for: A polymer scaffold for self-healing perovskite solar cells
Source: Nat Commun. 2016 Jan 6;7:10228. doi: 10.1038/ncomms10228 (PMC4729821; doi:10.1038/ncomms10228)
Supplement: Supplementary Information — Supplementary Figures 1-12 and Supplementary Note 1 [file ncomms10228-s1.pdf]

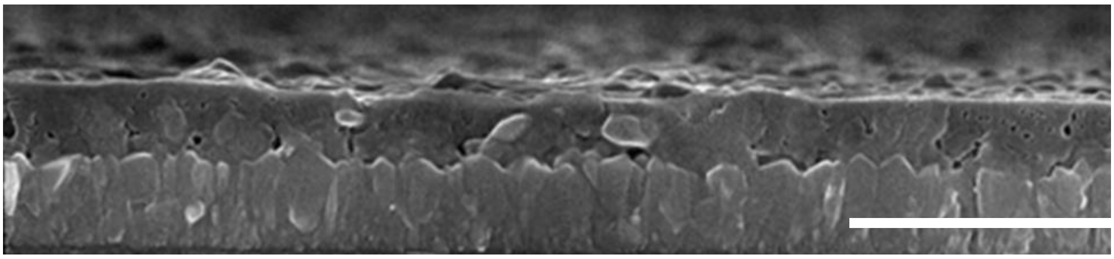

Supplementary Figure 1. *Cross-section SEM image of the polymer scaffold perovskite film using MAI:*PbI_2*=1:1 in DMF solvent on the FTO/glass substrate. Scale bar: 1* μm*.*

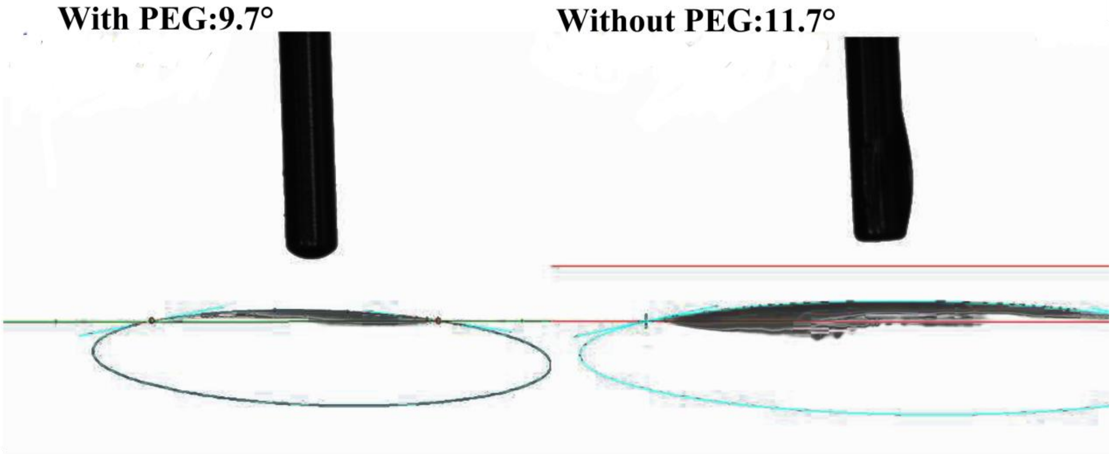

Supplementary Figure 2. *Contact angle measurement data of precursor solution with and without PEG. The smaller contact angle for the precursor solution containing PEG polymer chain (20 mg ml^{-1}) dipped on the FTO coated glass suggests a better wetting property. Four samples were measured and showed similar data.*

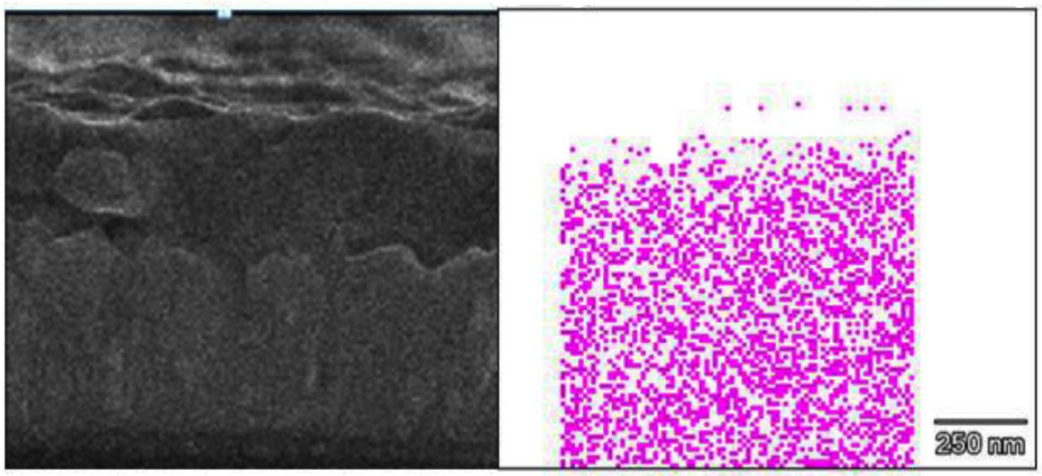

Supplementary Figure 3. *EDS mapping spectra of oxygen element distribution in the cross-section perovskite film spin-coated on the FTO glass, and its corresponding SEM image. Oxygen element stands for the PEG polymer distributed in perovskite film, considering only PEG molecule contains oxygen element. Scale bar: 250 nm.*

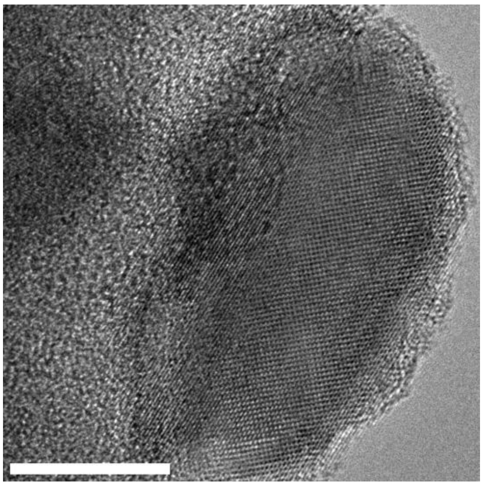

Supplementary Figure 4. *High resolution TEM image of perovskite crystals. Scale bar: 10 nm. The sample is prepared from perovskite precursor solution spin-coated on micro grid, then heated at 105°C/70 min. As can be seen from the HRTEM (High Resolution transmission electron microscopy), perovskite is partly coated by PEG molecule (amorphous phase), but the exposed area demonstrates a perfect crystal structure.*

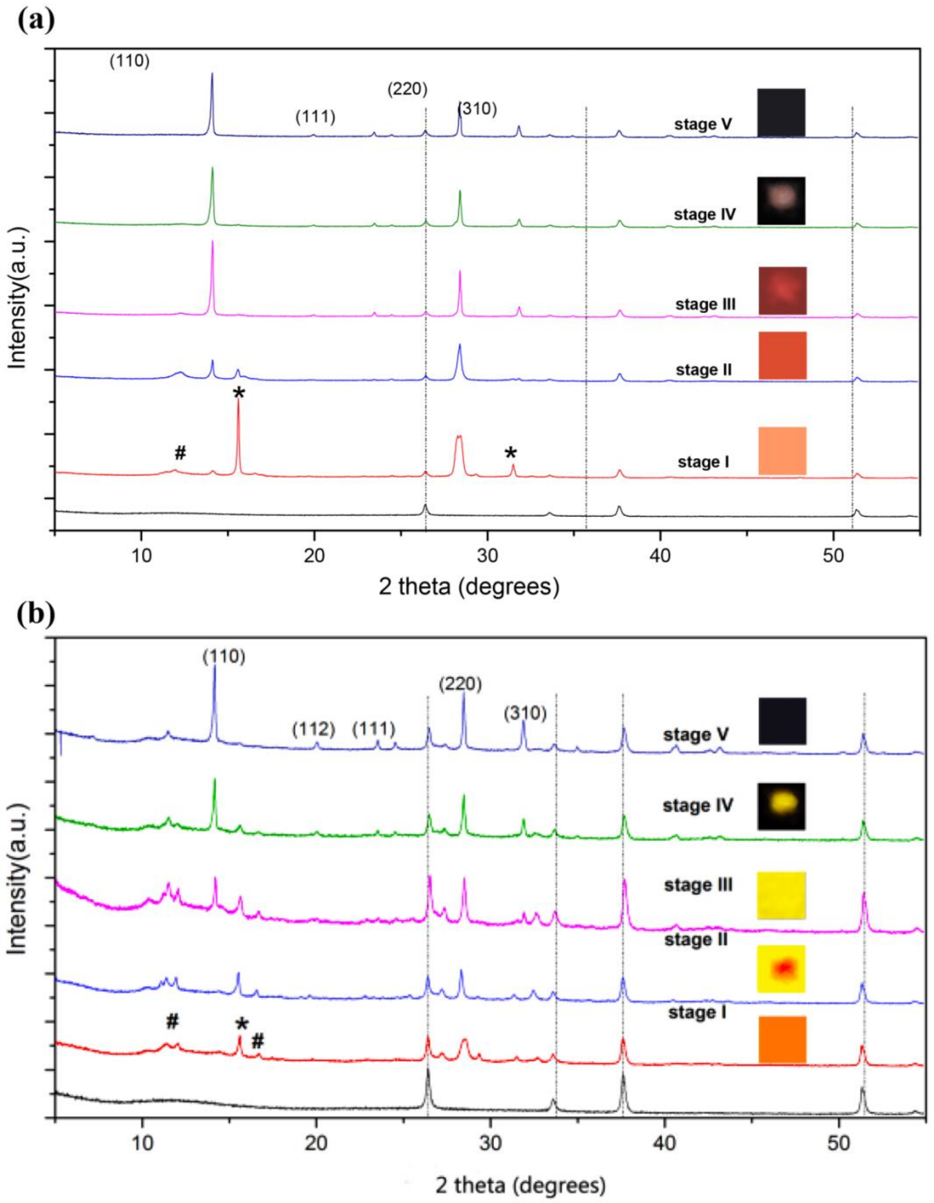

Supplementary Figure 5. *XRD patterns of perovskite thin film without PEG (a) and with PEG (20000 Da, 40 mgml^{-1}) (b) at different time stage under 100°C sintering, respectively, showing more details of the film formation evolution. Peaks indicated by # stands for intermediate phase and * stands for $MAPbCl_3$ phase. Stage I to V denotes to 60°C/30 min, 100°C/40 min, 100°C/70 min, 100°C/90 min, 100°C/110 min, respectively. The color squares represent the color change of the perovskite film at each corresponding stage.*

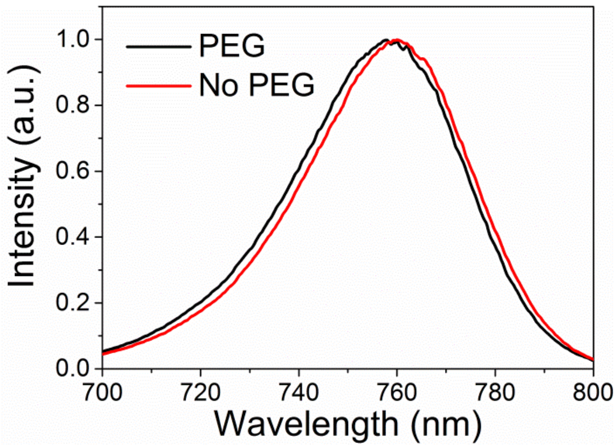

Supplementary **Figure 6.** *PL spectra of the perovskite film with and without PEG scaffold presents similar peak position and half-width of the band, further confirming the guaranteed crystal quality prepared from the precursor solution mixed with PEG polymer scaffold.*

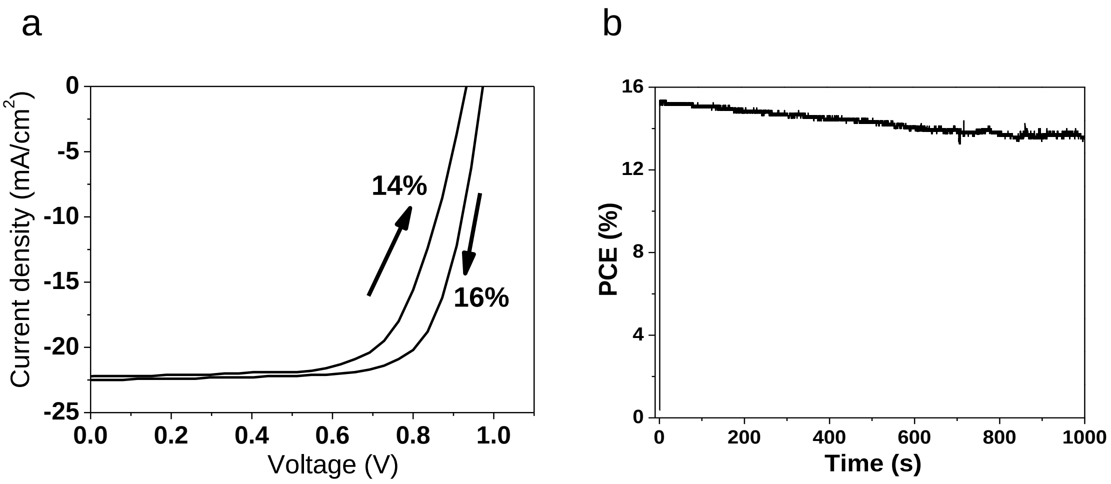

Supplementary Figure 7. *(a) J-V curve of the highest PCE 16% obtained from PPSC with optimal PEG molecule weight 20,000 Da and concentration 20 mg ml^{-1}, in forward and reverse scan under scan velocity of 500 mV s^{-1}. (b) Steady-state output of another representative device at 0.7 V bias. The falling behavior in steady-state output was improved by certain interfacial modification which had been worked out in our lab.*

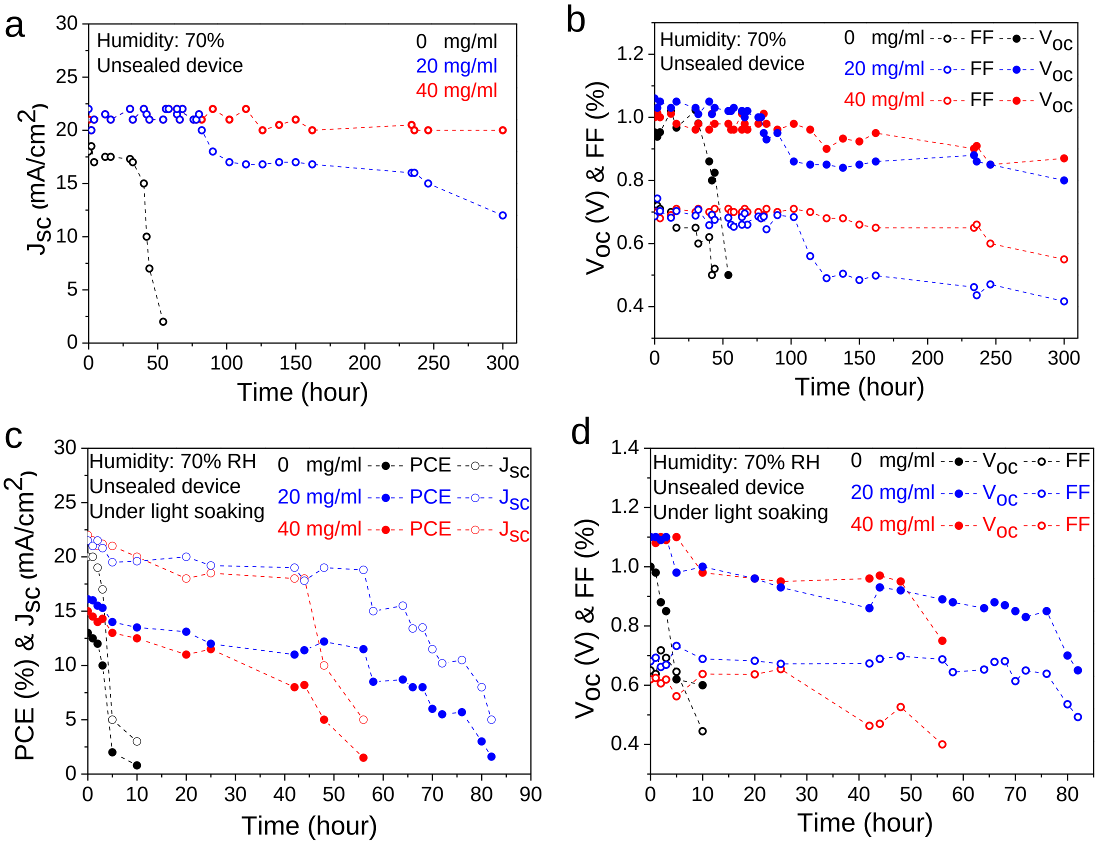

Supplementary Figure 8. *(a) J_{sc} of perovskite solar cells with 20/40 mg ml^{-1} PEG and without PEG scaffold exposed in high humid (~70% RH) dark environment without any sealing as a function of time. (b) V_{oc} and FF of perovskite solar cells with 20/40 mg ml^{-1} PEG and without PEG scaffold exposed in high humid (~70% RH) dark environment without any sealing as a function of time. (c) PCE and J_{sc} of perovskite solar cells with 20/40 mg ml^{-1} PEG and without PEG scaffold under continuous light illumination (70 $mWcm^{-2}$) in 70% humidity environment without any sealing. (d) V_{oc} and FF of perovskite solar cells with 20/40 $mgml^{-1}$ PEG and without PEG scaffold under continuous light illumination (70 $mWcm^{-2}$) in 70% humidity environment without any sealing.*

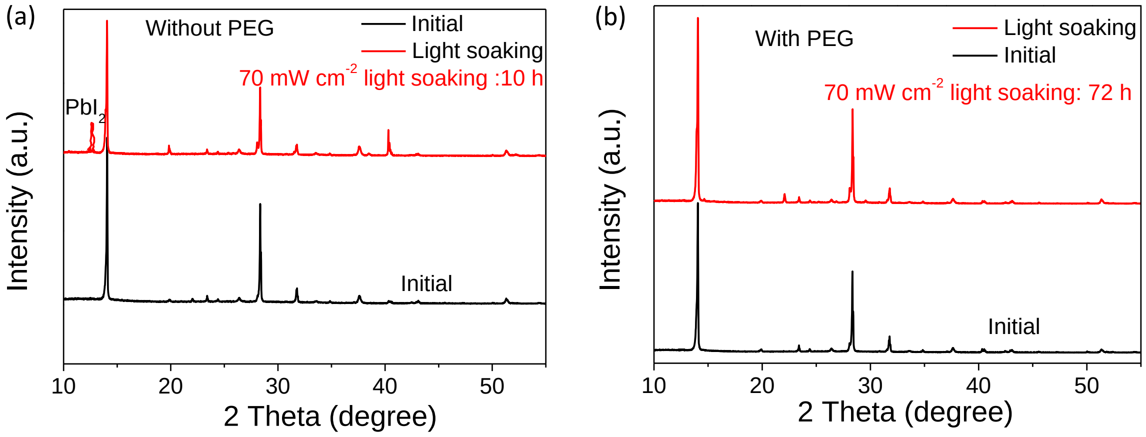

Supplementary Figure 9*. (a) XRD data from the initial perovskite film without PEG and that after continuous light soaking for 10 hours under 70 mW cm*$^{-2}$ *light intensity and humidity of 70%. (b) XRD data from the initial perovskite film with PEG scaffold and that after continuous light soaking for 72 hours under 70 mWcm*$^{-2}$ *light intensity and humidity of 70%, showing that perovskite film with PEG remain its crystal structure after light soaking.*

Based on XRD results above, the observed gradual decrease in PCE of PPSCs after 60 hours in stability test under light soaking (Supplementary Figure 8) was not caused by any decomposition or restructuring of bulk perovskite material. We conjecture that the bias-assisted light-generated charge traps located at interfaces may play a certain role.

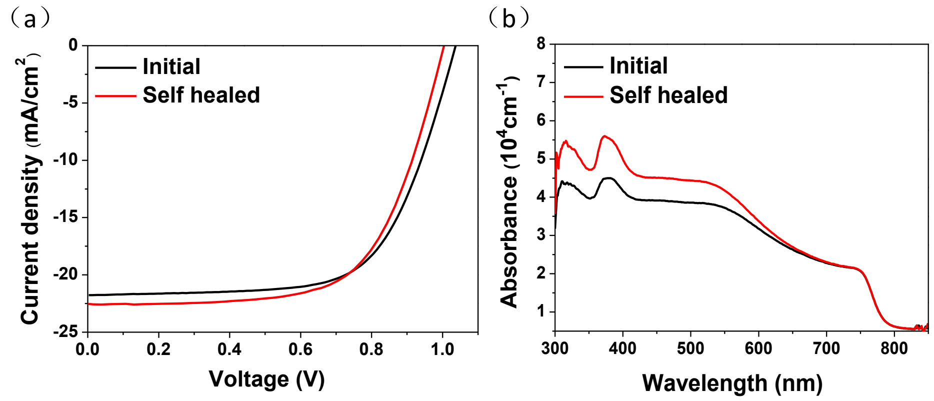

Supplementary Figure 10. *(a) J-V curves of PPSC devices fabricated by perovskite film with PEG scaffold after water vapor spray—self healing process and the initial film, respectively. The result shows that PPSC device fabricated by perovskite film with PEG scaffold after water vapor spray and self-healing showed almost unchanged J-V curve to that fabricated by its initial film without vapor spray. The slight increase of short-circuit current density is consistent with the overall improvement of optical density in perovskite film after vapor spray and self-healed (b), especially around the short wave band.*

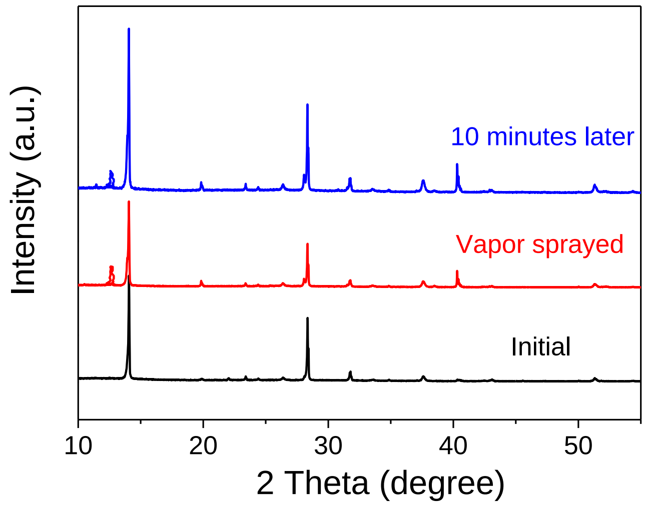

***Supplementary Figure 11**. XRD of perovskite film without PEG before and after water spray.*

In general case, followed by the reactive equation shown below:

$CH_3NH_3PbI_3 + 2H_2O \leftrightarrow CH_3NH_3PbI_3{\cdot}2H_2O \leftrightarrow PbI_2 + CH_3NH_3I{\cdot}2H_2O \leftrightarrow PbI_2 + CH_3NH_2{\cdot}H_2O + HI{\cdot}H_2O \leftrightarrow (light)\ PbI_2 + CH_3NH_2 + H_2O + (1/2)\ H_2 + (1/2)\ I_2 + H_2O$

the products except for PbI_2 are easy to evaporate into ambient atmosphere, inducing perovskite degradation irreversibly.

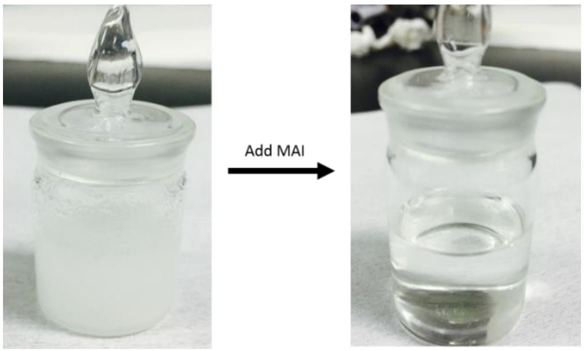

Supplementary Figure 12. *Photographs show that PEG can't dissolve in isopropanol (100 mg ml^{-1}) (turbid solution), but can dissolve rapidly by adding a small amount of MAI (1 mg ml^{-1}) (clear solution), indicating the strong interaction between PEG polymer and MAI.*

Supplementary Note 1.

It is for more detailed description for Supplementary Figure 5. It is found that the formation process with PEG polymer chains is different from the ones without PEG, indicating that the polymer PEG has a great effect on the growth dynamics. It is obvious that a longer time is needed for the perovskite formation with polymer scaffold, and the ultimate crystal is the perovskite from the (110)(220) patterns. As is reported, the diffraction angles 2θ at about 7, 12, 14, 17, 20, 28, 28.5, 31.8, 35, 40.5, 43 degree are ascribed to the crystal faces of the $CH_3NH_3PbI_3$ material (001) (100) (110) (002) (112) (044) (220) (310) (312) (224) (314), respectively. The unknown diffraction peaks at about 12° are contributed to the intermediate phase. PbI_2 peak is located at 12.8°, and $CH_3NH_3PbCl_3$ at 15.5°. After analyzing different phase at different stage, we put forward a self-consistent formation process and infer that the polymer scaffold has strong interaction with the CH_3NH_3Cl:

Stage I (without polymer scaffold): the intermediate phase forms and displays the light orange color, corresponding to the 12° peak in XRD patterns. During this stage, the perovskite forms at the same time, corresponding to the 14.14° peak.

Stage I (with polymer scaffold): however, the perovskite does not form at this stage from the absence of the (110)(220) peak, accompanied with a large amount of intermediate phase indicated by #.

Stage II (without polymer scaffold): with the release of DMF and CH_3NH_3Cl, the amorphous intermediate phase forms and the related CH_3NH_3Cl evaporates, leading to the formation of much more perovskite, which can be seen from the broad peak located at ~12° and the narrow peak at ~14°. At this stage, the color displays deep orange color.

Stage II (with polymer scaffold): with the release of DMF and little CH_3NH_3Cl, the intermediate phase crystallizes indicated by the narrowing of the peak at ~12°, because the polymer scaffold can prevent the CH_3NH_3Cl from evaporating, leaving time for the crystallization of the intermediate phase, displaying light yellow color with range fades away.

Stage III (without polymer scaffold): with the release of the byproduct CH_3NH_3Cl, the

reddish color is deepened with much more perovskite particles (black color) crystallized in the film, which can be clearly seen that the strong intensity located at $2\theta=14.14$ increases with prolonged time.

Stage III (with polymer scaffold): differing from the constituents without polymer scaffold at this stage, the color of the film turns from reddish brown into yellow completely, which is contributed to the phase transition of the intermediate phase because of the anchor of the CH_3NH_3Cl by the polymer's radical. After that, when the byproduct CH_3NH_3Cl releases slowly, the perovskites $CH_3NH_3PbI_3$ forms slowly, with the black circle appears on the yellow background.

Stage IV (without polymer scaffold): with further CH_3NH_3Cl released from both the intermediate phase and $CH_3NH_3PbCl_3$, which can be proved by the intensity decrease of the compounds in XRD spectrum, the conversion to the perovskite particle becomes much more completed and the color is reddish black

Stage IV (with polymer scaffold): with the time elongated, the CH_3NH_3Cl releases slowly, the perovskite phase forms at this stage, accompanied by the appearance of black color.

Stage V: when the excess CH_3NH_3Cl is completely consumed, the ultimate single phase $CH_3NH_3PbI_3$ forms, and this is the same for both cases.
